# Predicting Geographic Distribution of Forensically Significant Blow Flies of Subfamily Chrysomyinae (Diptera: Calliphoridae) in Northern Thailand

**DOI:** 10.3390/insects9030106

**Published:** 2018-08-21

**Authors:** Tunwadee Klong-klaew, Ratchadawan Ngoen-klan, Kittikhun Moophayak, Kom Sukontason, Kim N. Irvine, Jeffery K. Tomberlin, Pradya Somboon, Theeraphap Chareonviriyaphap, Hiromu Kurahashi, Kabkaew L. Sukontason

**Affiliations:** 1 Department of Parasitology, Faculty of Medicine, Chiang Mai University, Chiang Mai 50200, Thailand; somtunwa@gmail.com (T.K.); kom.s@cmu.ac.th (K.S.); pradya.somboon@cmu.ac.th (P.S.); 2 Department of Entomology, Faculty of Agriculture, Kasetsart University, Bangkok 10900, Thailand; ngernklun@yahoo.com (R.N.); faasthc@ku.ac.th (T.C.); 3 Nakhonsawan Campus, Mahidol University, Nakhonsawan 60130, Thailand; khun_khithop@hotmail.com; 4 National Institute of Education, Nanyang Technological University, 50 Nanyang Avenue, Singapore 639798, Singapore; kim.irvine@nie.edu.sg; 5 Department of Entomology, Texas A&M University, 2475 TAMU, College Station, TX 77843, USA; jktomberlin@tamu.edu; 6 Department of Medical Entomology, National Institute of Infectious Diseases, Tokyo 162-8640, Japan; MLB15110@nifty.com

**Keywords:** spatial distribution, predictive distribution, GIS, forensic entomology

## Abstract

Blow flies (Diptera: Calliphoridae) are carrion-breeding flies that are commonly used as evidence in forensic investigation. An adequate knowledge of ecological and geographical data of blow fly has a direct application in forensic science, as far as estimating time of colonization or corpse relocation. The aim of this study was to evaluate the occurrence of four species of Chrysomyinae (*Chrysomya pinguis*, *Chrysomya chani*, *Chrysomya villeneuvi*, and *Ceylonomyia nigripes*) across six land use types in central Chiang Mai, northern Thailand. Eighteen study sites were selected for sampling across three districts of Chiang Mai province (Mueang Chiang Mai, Mae Rim, and Hang Dong). Adult flies were collected every two weeks using a funnel trap baited with 1-day tainted beef offal. The predicted geographic distributions of forensically important blow fly species were modeled using the computer program ArcGIS, based on selected climatic variables (temperature, relative humidity, and light intensity) recorded at study sites. During the study period, 1298 adult flies were collected, with peak fly occurrence during summer (April–May). Seasonal fluctuation patterns varied depending on fly species. Climatic factors displayed diverse impact on associated fly populations. Identified species were restricted mainly to mixed deciduous forests (MDF) especially in the mountainous area. None of these flies were trapped in an urban area.

## 1. Introduction

Many blow flies (Diptera: Calliphoridae) species are carrion-breeding and have veterinary, medical, and forensic importance [1,2]. They play a prominent role as vectors of human as well as livestock, pathogens [1,2]. Additionally, they are currently the most commonly used arthropod group in forensic entomology research to determine time of colonization as related to the post-mortem interval (PMI_min_) as they are commonly associated with decomposing human remains [3,4]. The presence and abundance of such species are useful for reconstructing crime scenes, as particular carrion-associated species have different environmental requirements, therefore they colonize carcasses in different habitats [5].

In Thailand, the vast majority of forensic important species investigations have been focused on species identification [6,7,8], developmental rate of immature stage [9,10], and insect succession [11,12]. Furthermore, fly surveys have been conducted on a local scale or limited to only specific land use types, for instance, in forest or in mountainous area [13,14]. Knowledge about the spatial and temporal distributions is still lacking. There is very little literature on the geographic distribution of forensically important flies in Thailand, and published studies focused on the common species (e.g., *C. megacephala*, *C. rufifacies*, and *M. domestica*) [15,16].

The objective of this study was to evaluate the occurrence of Chrysomyinae (Diptera: Calliphoridae) across a variety of land use types in central Chiang Mai, northern Thailand. Four species of blow fly in the subfamily Chrysomyinae were chosen because they are among the most prominent groups of insects associated with corpses and carrion, and are therefore important forensic indicators. Furthermore, they have been reported from human corpses in many countries [17,18,19,20]. To utilize these flies as evidence in forensic investigations, one needs to know where they occur. Blow flies are ectotherms, temperature heavily influences their development, behavior, and physiology [21]. But the influence of temperature and other climatic factors (relative humidity and light intensity) on their geographic distribution is unknown. In this study, the relationship of fly abundance with climatic factors was studied. Maps of predicted distributions for four species of carrion-associated Chrysomyinae were constructed using ArcGIS program.

## 2. Materials and Methods

### 2.1. Study Areas

**Figure 1:**
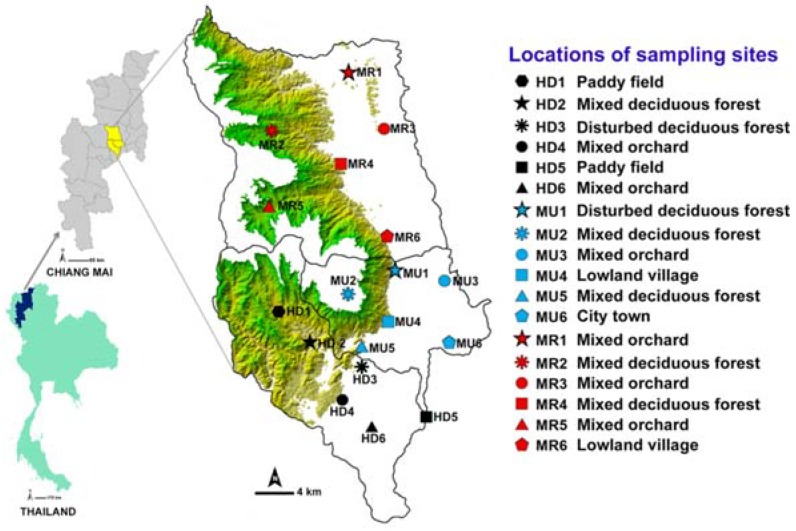
Map of Thailand showing three sample districts (Mueang Chiang Mai, MU; Mae Rim, MR; and Hang Dong, HD) and 18 sampling locations. Green shade represents a mountainous area.

This study was conducted in three districts: Urban Mueang Chiang Mai (MU), suburban Hang Dong (HD), and Mae Rim (MR) in the central part of Chiang Mai province (Figure 1). The entire area of each of the three districts was stratified initially based on a systematic random sampling method [22]. The contour maps of Chiang Mai province (MapMagic^TM^ scale 1:150,000 with a UTM projection type, Everest Spheroid and the Indian 1975 Datum) were used for plotting the study area. The description of study sites selection and land use categories was provided in the previous study [16]. Six different land uses were classified: disturbed mixed deciduous forest (DDF), mixed deciduous forest (MDF), mixed orchard (MO), paddy field (PF), lowland village (LLV), and city town (CT) [15,16].

### 2.2. Fly Collection

Sampling was conducted at 18 sites, representing six land uses and covers three districts of the central part of Chiang Mai province, Thailand [16]. The collection was performed from May 2009 to May 2010. Adult fly collection was carried out every two weeks using an in-house prototype reconstructable funnel trap kit. The collection procedure and description of the trap was described previously [15,16]. Two hundred and fifty grams of 1-day tainted beef offal [23] was used as bait positioned underneath the fly entrance module at the base of trap. The traps were exposed for a 1-h period between 9:00 and 12:00 am. The physical variables were noted at each study site, including temperature (°C), relative humidity (%) (Digital Hygro-Thermo Meter (DHT-1); (Daeyoon Scale Industrial Co., Ltd., Seoul, South Korea) and light intensity (lux) (LUX/FC light meter TM-204 Tenmars, Tenmars Electronics Co., Ltd., Taipei, Taiwan). The GPS coordinates were recorded using Garmin ^TM^ eTrex Handheld GPS (Garmin China Co., Ltd., Chaoyang, China).

All trapped flies were sacrificed using a freezer set at 0 °C for 2 h. They were identified individually under a dissecting microscope (model SZ2-ILST, Olympus Corporation, Tokyo, Japan) using taxonomic keys of Tumrasvin et al. [24], sexed and counted at the laboratory of the Department of Parasitology, Faculty of Medicine, Chiang Mai University. Four species of Chrysomyinae flies were studied, comprising: *Chrysomya pinguis* (Walker), *Chrysomya chani* Kurahashi, *Chrysomya villeneuvi* Patton, *Ceylonomyia nigripes* (Aubertin). The adults of these species are metallic green in coloration, presenting setulae on the posterodorsal surface of stem-vein of wings. Prealar knobs bear erect hairs. The adult of *Cey. nigripes* is a small size blow fly (4.0–6.0 mm body size). It has white mesothoracic spiracles. Eyes are dichoptic in both sexes. An orientation of sternopleural bristles is formed as 0 + 1 pattern. The adults of *C. villeneuvi*, *C. chani* and *C. pinguis* have fuscous gena and brown to black mesothoracic spiracles. Femora are greatly swollen in *C. villeneuvi* and the disc of the 5th tergite of males is covered with dense short hairs but almost bare in female. In *C. chani* and *C. pinguis*, the disc of the 5th tergite is covered with many fine erect bristles in both sexes. The adult of *C. chani* has a white anterior half, with yellowish-white margin, of upper squama. On the contrary, in *C. pinguis*, an anterior half of upper squama is covered with dark brown to black hairs.

### 2.3. Statistical Analysis

For the statistical and geospatial analyses, a base-10 logarithm (log_10_) transformation of the raw data (*n* + 1) was applied to improve the normality of distribution. Bivariate correlation analysis and Pearson Product Moment Correlation (*r*) were analyzed to measure the association between climatic variables (temperature, relative humidity and light intensity) and fly populations. The data for all study sites were combined before analysis. One-way analysis of variance (ANOVA) followed by a post-hoc Bonferroni test (homogeneity of variance: *p* > 0.05) or Dunnett T3 test (homogeneity of variance: *p* < 0.05) were employed to compare mean total number of flies between land use types using SPSS 22.0 (IBM Corp., Armonk, NY, USA) for Windows (*α* = 0.05). When the significant relationships were found, the kriging/co-kriging techniques were used to model spatial patterns of flies in the different localities using the Geostatistical Analyst tool of ArcGIS 9.2 program (ESRI, Redlands, CA, USA). These techniques help to predict values at unsampled locations [16]. In this study, the parameters were used in co-kriging including climatic factors that had a significant relationship with fly numbers and the total number of flies collected during May 2009 to May 2010. Furthermore, the “land use” categories were also included in the analysis as dummy variables. The mathematical model, which provided the lowest root-mean-square prediction error, was chosen as a model for estimating the semivariogram/covariance function [16].

## 3. Results

**Figure 2:**
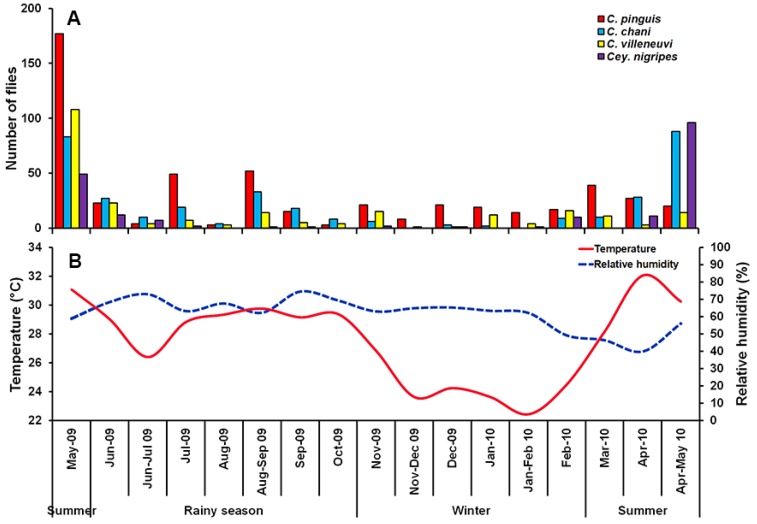
Monthly fluctuations in population density of *C. pinguis*, *C. chani*, *C. villeneuvi*, and *Cey. nigripes* determined using a reconstructable funnel trap baited with 1-day tainted beef offal in Chiang Mai province, northern Thailand, May 2009 to May 2010 (**A**), and Annual variation of temperature and relative humidity recorded during the fly survey (**B**).

A total of 1298 Chrysomyinae flies were collected, comprising four species: *C. pinguis*, *C. chani*, *C. villeneuvi*, and *Cey. nigripes*. The most abundant fly was *C. pinguis* (*n* = 512; 39.4%), followed by *C. chani* (*n* = 348; 26.8%), *C. villeneuvi* (*n* = 245; 18.9%), and *Cey. nigripes* (*n* = 193; 14.9%). These species were collected mostly in MDF (75.6%), followed by MO (11.5%), PF (10.9%), LLV (1.4%) and DDF (0.7%) (Table 1). None of these flies were trapped in CT throughout a one-year survey. Seasonal fluctuations of collected flies were found, with a peak in the summer (late April-May). The reduction of trapped specimens was observed in the rainy season and remained constantly low through the winter (Figure 2).

**Figure 3:**
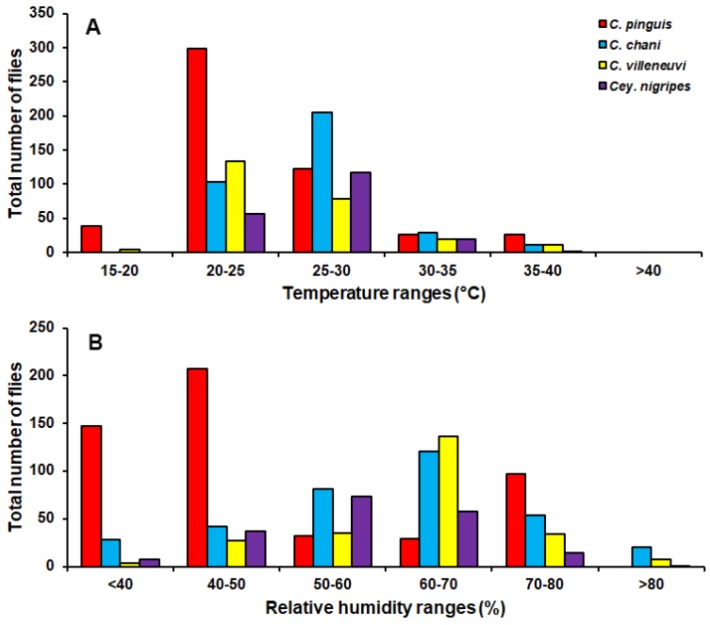
Total number of flies captured at different temperature (**A**) and relative humidity ranges (**B**).

**Figure 4:**
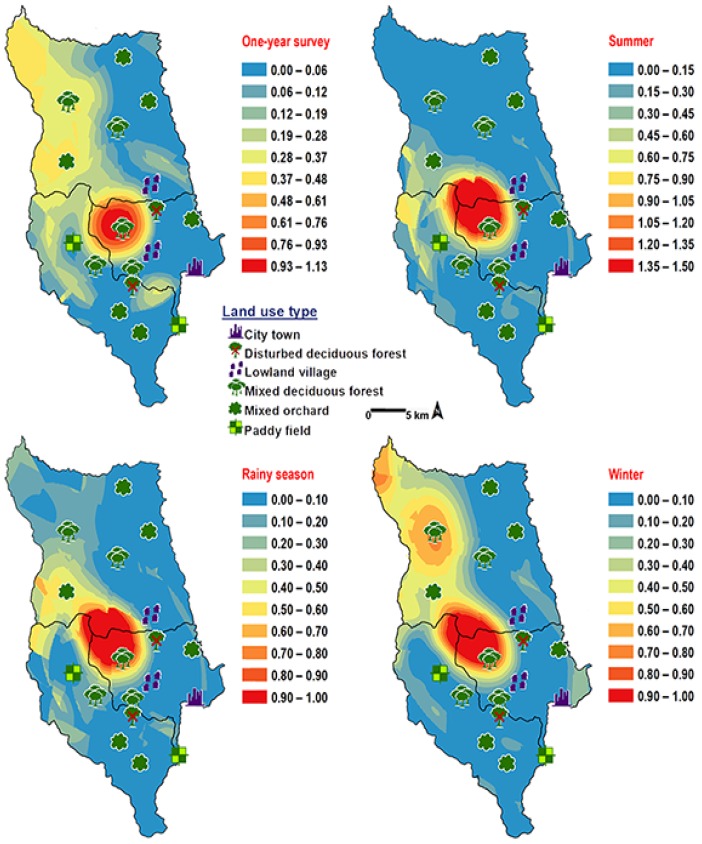
Predictive distribution maps of *Chrysomya pinguis*. The red areas symbolize the highest density of flies calculated by the analysis, while the blue areas indicate the lowest density. The scale corresponds to the natural logarithm of (fly density +1).

*Chrysomya pinguis* exhibited a preference for MDF. No flies were collected in DDF and CT (Table 2). The abundance of flies was weak negatively correlated with light intensity (*r* = −0.319, *p* = 0.0001) and temperature (*r* = −0.232, *p* = 0.0001), but showed no correlation with humidity (*r* = 0.051, *p* = 0.374) (Table 3). High number of this species was collected at 20–25 °C and 40–50% RH (Figure 3). Co-krigged prediction maps were produced using fly population data, land uses, temperature, and light intensity. This species can probably be found predominantly in MDF at high altitude (MU2; 950 m asl.). The seasonal maps also showed a similar pattern to a year-survey map (Figure 4).

**Figure 5:**
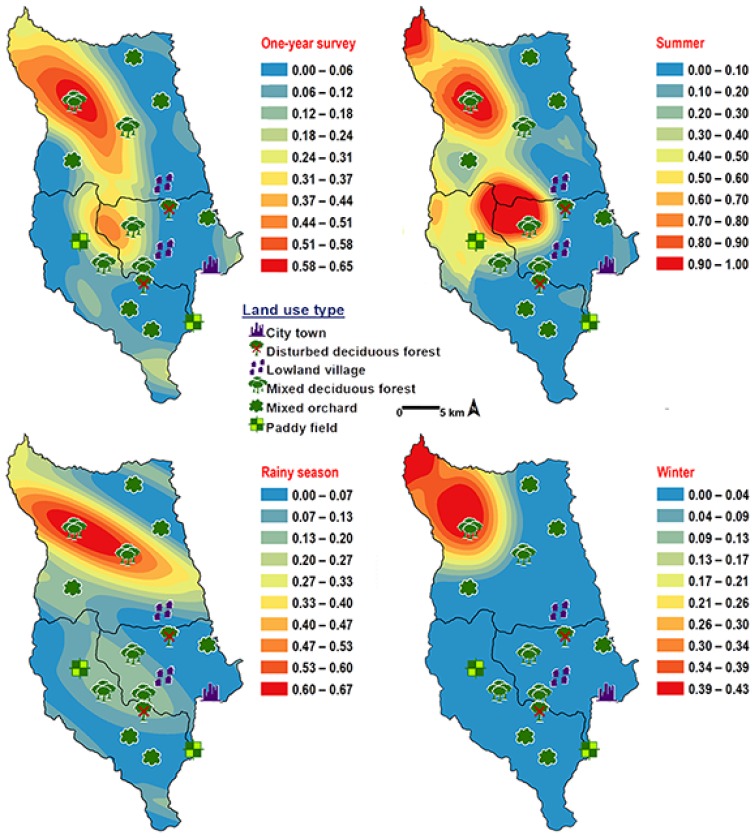
Predictive distribution maps of *Chrysomya chani*. The red areas symbolize the highest density of flies calculated by the analysis, while the blue areas indicate the lowest density. The scale corresponds to natural logarithm of (fly density +1).

*Chrysomya chani* had quite similar results to that of *C. pinguis*. This species was prevalent in MDF (Table 2). The abundance of *C. chani* showed no correlation with any climatic factors (Table 3). High number of this species was trapped at 25–30 °C and 60–70% RH (Figure 3). A distribution modelling was created using specimens collection data and land use types. According to predicted geographical distribution, this species can probably be found in MDF at 407 m asl. in Mae Rim district throughout the year. The seasonal prediction maps represented large number of *C. chani* in MDF of mountainous area. Interestingly, in summer this species can probably be found in MDF at 950 m. asl. (MU2) (Figure 5).

**Figure 6:**
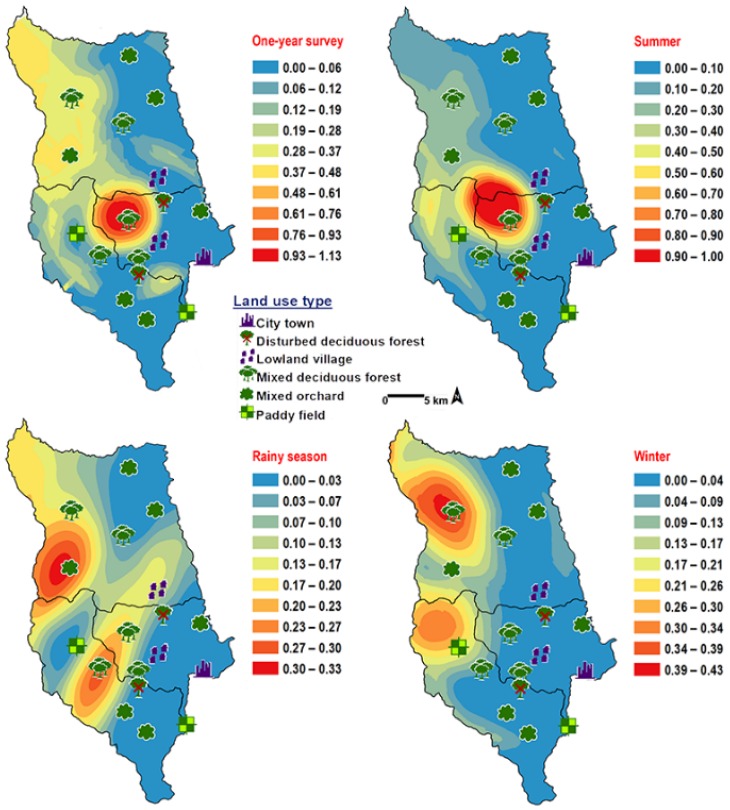
Predictive distribution maps of *Chrysomya villeneuvi*. The red areas symbolize the highest density of flies calculated by the analysis, while the blue areas indicate the lowest density. The scale corresponds to natural logarithm of (fly density +1).

*Chrysomya villeneuvi* was prevalent in MDF and PF (Table 2). The abundance of *C. villeneuvi* showed weak negative correlation with light intensity (*r* = −0.180, *p* = 0.002) but had no correlation with temperature and relative humidity (Table 3). However, high number of this species was collected at 20–25 °C and 60–70% RH (Figure 3). The prediction maps were generated by combining data of fly population, light intensity, and land use types in the analysis. From a year-survey map, the abundance of this species was predicted in MDF at 950 m asl. (MU2). In summer, the similar pattern to a one-year survey was observed. In rainy season, high number of *C. villeneuvi* was predicted in MO and MDF in mountainous area. In winter, this species was predicted largely in MDF and paddy field (Figure 6).

**Figure 7:**
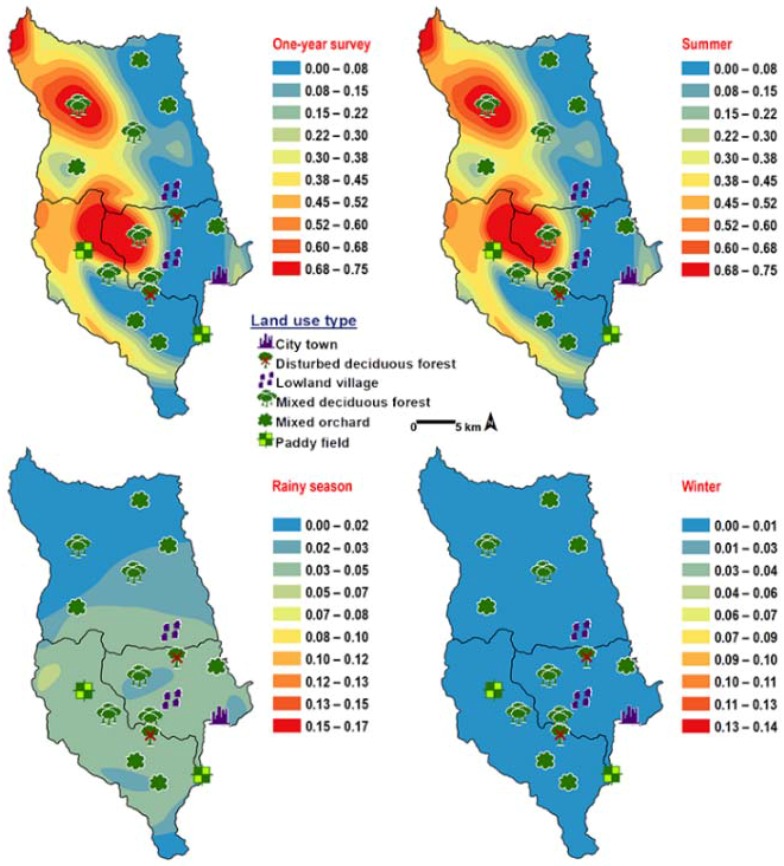
Predictive distribution maps of *Ceylonomyia nigripes*. The red areas symbolize the highest density of flies calculated by the analysis, while the blue areas indicate the lowest density. The scale corresponds to natural logarithm of (fly density +1).

*Ceylonomyia nigripes* was collected widely in all land uses except for the city (Table 2). No significant relationship was found among the abundance of this species and climatic factors (Table 3). High number of flies was collected at 25–30 °C and 50–60% RH (Figure 3). Fly population data and land uses were applied to create the krigged prediction maps. A one-year survey and summer prediction maps represented similar pattern, as this species was predicted largely in MDF and other land uses along the mountainous area. Low numbers of this species were predicted in the rainy season and in winter (Figure 7).

In summary, four species of Chrysomyinae reported here were most abundant in mixed deciduous forest especially in mountainous areas. However, none of these flies were collected in the city town. Although high numbers were collected in the summer months, variations in fluctuation patterns were dependent upon flies’ species. Climatic factors showed different impact on flies’ population. Nonetheless, none of these flies was collected when the temperature was >40 °C (Figure 3).

## 4. Discussion

As previously stated, an adequate knowledge of ecology and geographical abundance of blow flies has a direct application in forensic science [25]. However, the information on geographic distribution of forensically important flies in Thailand is limited. This study was the first to determine the abundance of four forensically important Chrysomyinae flies; *C. pinguis*, *C. chani*, *C. villeneuvi*, and *Cey. nigripes*, over a full year in northern Thailand. Additionally, predictive distribution maps of these species were generated using systematic random sampling and GIS. Although the collection sites in this study were chosen in different broad ecological locations, our results clearly indicate these four species prefer mixed deciduous forest over the residential areas (e.g., city town and lowland village). This is consistent with the previous study, in which *C. chani* and *Cey. nigripes* were collected mostly from corpses found in the forest [19]. A recent study also documented *C. pinguis* and *C. villeneuvi* associated with a human corpse found in forest of high mountainous area (1200 m asl.) in northern Thailand [26]. However, *C. villeneuvi* was also reported in a case from urban/suburban area [19]. In Australia, *Cey. nigripes* was strongly associated with areas of high percentage of forest cover [27]. On the contrary, the study in Malaysia reported *Cey. nigripes* on the remains found in a house/apartment [20]. Therefore, the variation of fly infestations in each locality could be due to the availability of food sources, food preferences, ecological features of each locality, and the disturbances of humans.

Local availability of food sources could possibly be the factor affecting the abundance of blow flies found in each land use. In natural conditions, blow flies are typically necrophagous and saprophagous. As bacteria decompose, the necrophagous flies feed on removing the soft tissues of animal carcasses [28]. These four species might prefer small to large wild animal carcasses available in forest areas rather than the garbage or small carcasses, which may be the main resources in nearby residential areas. Furthermore, ecological features in each land uses probably affected ambient temperature as a result of differences in exposure of sunlight. The degree of sun exposure was influenced by the denseness of the trees, the increases in tree cover as in the forest increase sun protection that maintains the temperature range [29]. The study in Malaysia reported a greater variation in weather data recorded in the rural area than in the forest [30]. Furthermore, fewer disturbances from humans in mixed deciduous forests might be another factor affecting these flies’ abundance. As reported in Japan, *C. pinguis* larvae were more likely to be found on corpses found in the less populated areas [17].

Seasonal variation impacted the abundance of blow flies. When individual species were examined in different habitats across seasons, we determined that the abundance and distribution of the fly species in question were impacted. According to our study, blow fly abundance peaked during summer, and diminished in the rainy season and winter. In general, this finding is consistent with previous reports, as greater numbers of flies were found in hot weather season (23–45.2 °C) than in cold weather season (16.7–34.9 °C) [3,25,31]. The higher temperatures in the summer period accelerate the developmental rate of blow flies. Conversely, the lower temperatures (16.7–20 °C) in cold weather conditions reduce the attractiveness of the baited trap [32] and the flying activity of flies (Figure 3). Our results further indicate only *C. pinguis* and *C. villeneuvi* were active throughout the year. However, it should be noted that these two species were trapped mostly at temperatures from 20–25 °C. The reduction in *C. chani* and *Cey. nigripes* numbers during the rainy season and/or winter most likely is due to their preference for higher temperatures (25–30 °C). A study in China also suggested *Cey. nigripes* prefers warmer temperatures [33]. However, conflicting results were observed by Moophayak et al. [13], when *Cey. nigripes* only vanished from the summer collections. This may be due to differences in study designs (e.g., sampling method, collection period, and study sites) or population variation [34]. Furthermore, the attractiveness of 1-day tainted bait used in this study may not be suitable for luring *C. chani* and *Cey. nigripes*, as they prefer an advanced state of decomposition [35,36]. Especially during the rainy season and the winter, volatiles released by the bait most likely decreased thus reducing attraction of flies.

Environmental factors undoubtedly impacted the diversity of blow flies, however, differences were observed among the species. Assessment of climatic factors on fly populations indicated *C. pinguis* increased when temperature (20.7–23.4 °C) and light intensity (630–1870 lux) were low. A previous laboratory study indicated *C. pinguis* was more active at 15 °C, which supports its preference of the areas and seasons with low temperature [37]. Light intensity also had a negative effect on the population of *C. villeneuvi*. These may support the habitat preferences of these two species, because a mixed deciduous forest has a dense tree cover that protects from the sun and maintain temperature. According to the statistical analysis, *C. chani* and *Cey. nigripes* showed no correlation with any climatic factors. Furthermore, humidity has no influence on any species in this study. Azevedo and Krüger [38] reported *Chrysomya* spp. have a positive correlation with temperature; humidity was determined not to be a factor. Our results clearly indicate that flies are not active when temperature exceeds 40 °C. Similar results were determined for *C. rufifacies* in Texas, USA with flies not being active during the hottest period of the day [39]. Because blow flies are ectotherm, the temperature is a major factor limiting their environmental preferences [21]. A study in Taiwan also reported that a significantly lower eclosion ratio of *C. pinguis* than *C. megacephala* at 30 °C indicated differences between temperature adaptations [37]. Therefore, temperatures >40 °C may be too high for these forest-associated species to be adapted. However, it should be emphasized that climatic factors (e.g., temperature, relative humidity and light intensity) in this study were recorded as point data collection at the onset of fly trap setting. Therefore, using climate data logger to record data over the study period may provide a more accurate relationship of environmental factors on fly population dynamics.

The species distribution modelling can identify the preference of a particular species in a certain locality. Therefore, it could be applied to verify the potential of corpse relocation in a forensic scenario [40]. Human remains are more likely to be moved from an open area to a forest habitat because it is more easily to be concealed. As a result, the insects which inhabit a corpse in an open environment but not in forested habitats might be good indicators of remains being relocated [41]. Our results clearly indicated these four Chrysomyinae species prefer mixed deciduous forests over the residential areas (e.g., city town and lowland village). Consequently, these four species might not be suitable indicators for corpse relocation when compare with the common blow flies, *C. megacephala* and *C. rufifacies* [15,16]. However, the maggots of theses flies can be used in the estimation of PMI_min_ when corpses are found in the forest. This study was carried out in only one urban area (Mueang Chiang Mai district). Therefore, the absence of a particular species in a specific habitat does not make it unsuitable to be used as an insect evidence to explain the circumstances associated to death.

During the early postmortem period, the PMI_min_ estimation was calculated based on the evaluation of the presumed oldest maggot found on the corpses. As developmental time is species specific, proper identification of the larvae found on a corpse is crucial for accuracy in age estimation [4]. Although larval morphology of these four Chrysomyinae has been well studied, they share some common features making identification difficult (e.g., *C. megacephala* and *C. rufifacies*) [26,42,43,44]. Even though, DNA-based techniques can be used for species identification, there are some limitations. For instance, the molecular approach relied on mitochondrial genes were unable to differentiate *C. pinguis* and *C. megacephala* [45]. In India, *C. chani* and *C. megacephala* were unable to be distinguished using the COI gene [46]. Our results clarified the distribution ranges of *C. pinguis* and *C. chani*, which are different from *C. megacephala* [16]. Thus, when corpses were found in mixed deciduous forest in mountainous area in Thailand, four Chrysomyinae flies reported here are the major species that should be taken into account.

## 5. Conclusions

This study provides greater detail of the distribution of four carrion-associated species and the factors influencing their distribution. This research generates a better understanding of the distribution of these forensically important fly species in Thailand. Nonetheless, further studies on biological aspects (e.g., biology, life table, insect succession) of these species associated to different land uses, are necessary to gain better understanding on the potential of these species as entomological evidence in forensic investigations.

## Figures and Tables

**Table 1:** Climatic factors (temperature, relative humidity, and light intensity) recorded and total numbers of Chrysomyinae flies collected at each land use types.

Land Uses	Climatic Factors Recorded *			Number of Chrysomyinae Flies Collected				
	Temperature (°C)	Relative Humidity (%)	Light Intensity (Lux)	*C. pinguis*	*C. chani*	*C. villeneuvi*	*Cey. nigripes*	Total
mixed deciduous forest	26.5 (16.7–39.8)	67.0 (35–87)	26,550 (193–107,500)	417	282	163	119	981
disturbed mixed deciduous forest	28.4 (21.0–39.3)	61.5 (35–80)	59,403 (2320–359,600)	0	2	2	5	9
mixed orchard	28.4 (19.7–45.2)	59.0 (25–89)	47,200 (1000–118,500)	83	18	27	21	149
paddy field	27.5 (19.6–40.8)	63.5 (39–83)	36,300 (6700–112,000)	11	44	44	42	141
lowland village	28.3 (18.7–35.8)	65.0 (36–83)	38,550 (2900–95,700)	1	2	9	6	18
city town	25.8 (18.8–30.7)	64.0 (40–78)	22,000 (442–94,000)	0	0	0	0	0

* Median (min–max).

**Table 2:** Mean number of Chrysomyinae flies collected based on land use types in Chiang Mai province, May 2009–May 2010.

Land Uses	*n*	Fly Species			
		*C. pinguis*	*C. chani*	*C. villeneuvi*	*Cey. nigripes*
mixed deciduous forest	84	4.96 ± 1.88 ^a^	3.36 ± 0.91 ^a^	1.94 ± 0.04 ^a^	1.42 ± 0.71 ^a^
disturbed mixed deciduous forest	34	0	0.06 ± 0.04 ^b^	0.06 ± 0.04 ^b^	0.15 ± 0.10 ^a^
mixed orchard	101	0.82 ± 0.35 ^b^	0.18 ± 0.07 ^b^	0.27 ± 0.12 ^b^	0.21 ± 0.12 ^a^
paddy field	34	0.32 ± 0.19 ^b^	1.29 ± 1.20 ^b^	1.29 ± 0.90 ^a,b^	1.24 ± 1.14 ^a^
lowland village	34	0.03 ± 0.03 ^b^	0.06 ± 0.06 ^b^	0.26 ± 0.16 ^b^	0.18 ± 0.08 ^a^
city town	15	0	0	0	0

Data are presented as mean number ± SE; *n* a frequency shows the number of observations in each land uses. Different letter (a,b) shown as a significant difference within groups (ANOVA: *p* < 0.05).

**Table 3:** Correlation coefficient between the climatic factors and fly populations in the central area of Chiang Mai province, May 2009–May 2010.

Climatic Factors		Fly Species			
		*C. pinguis*	*C. chani*	*C. villeneuvi*	*Cey. nigripes*
temperature	*r*	−0.232 **	−0.003	−0.83	0.017
	*p*	0.000	0.961	0.148	0.768
relative humidity	*r*	0.051	0.064	0.086	−0.042
	*p*	0.374	0.266	0.138	0.462
light intensity	*r*	−0.319 **	−0.102	−0.180 **	0.016
	*p*	0.000	0.078	0.002	0.779

The values are presented as Pearson correlation coefficient (*r*) (*P*-sig, two-tailed). * *p* = 0.05, ** *p* = 0.01, significant correlations.
